# Laser-Evoked Vertex Potentials Predict Defensive Motor Actions

**DOI:** 10.1093/cercor/bhv149

**Published:** 2015-08-06

**Authors:** M. Moayedi, M. Liang, A. L. Sim, L. Hu, P. Haggard, G. D. Iannetti

**Affiliations:** 1 Department of Neuroscience, Physiology and Pharmacology, University College London, London WC1E 6BT, UK; 2 School of Medical Imaging, Tianjin Medical University, Tianjin 300203, China; 3 Key Laboratory of Cognition and Personality (Ministry of Education) and School of Psychology, Southwest University, Chongqing 400715, China; 4 Institute of Cognitive Neuroscience, Department of Psychology, University College London, LondonWC1N 3AR, UK

**Keywords:** agency, defense, event-related potentials, saliency, sensorimotor integration, threat detection

## Abstract

The vertex potential is the largest response that can be recorded in the electroencephalogram of an awake, healthy human. It is elicited by sudden and intense stimuli, and is composed by a negative–positive deflection. The stimulus properties that determine the vertex potential amplitude have been well characterized. Nonetheless, its functional significance remains elusive. The dominant interpretation is that it reflects neural activities related to the detection of salient stimuli. However, given that threatening stimuli elicit both vertex potentials and defensive movements, we hypothesized that the vertex potential is related to the execution of defensive actions. Here, we directly compared the salience and motoric interpretations by investigating the relationship between the amplitude of laser-evoked potentials (LEPs) and the response time of movements with different defensive values. First, we show that a larger LEP negative wave (N2 wave) predicts faster motor response times. Second, this prediction is significantly stronger when the motor response is defensive in nature. Third, the relation between the N2 wave and motor response time depends not only on the kinematic form of the movement, but also on whether that kinematic form serves as a functional defense of the body. Therefore, the N2 wave of the LEP encodes key defensive reactions to threats.

## Introduction

The appropriate detection and reaction to potentially threatening events in the sensory environment is critical to survival. Sudden and intense sensory stimuli elicit a large, negative–positive biphasic wave in the human electroencephalogram (EEG), maximal at the scalp vertex (the vertex potential; Bancaud et al. 1953; Walter 1964). This large vertex response is a specific subset of event-related potential components, and represents the largest synchronization of neural activity that can be recorded from the scalp of an awake, healthy individual. It can be elicited by stimuli of virtually all sensory modalities, provided that they are salient (Walter 1964; Mouraux and Iannetti 2009). When elicited by either innocuous or noxious somatosensory stimuli, the negative–positive vertex wave is labeled as N2–P2 (Treede et al. 1988).

The magnitude of the vertex potential is sensitive to changes in some stimulus attributes, but not in others. For example, increases in the energy of a somatosensory stimulus increase N2 amplitude, whereas decreases in stimulus energy and small displacements have little effect (Torta et al. 2012; Ronga et al. 2013). Therefore, it has been posited that the N2 wave of the vertex potential reflects the detection of potential threats in the sensory environment (Ritter et al. 1968; Fruhstorfer 1971; Legrain et al. 2011; Valentini et al. 2011; Ronga et al. 2013).

Nonetheless, despite extensive characterization of the stimulus properties that elicit vertex potentials and influence their amplitude, as well as their brain generators (Mouraux and Iannetti 2009), it is not known whether and how a vertex potential is useful. In cognitive psychophysiology research, the dominant interpretation is based on salience: The vertex potential reflects the detection of salient stimuli in the sensory environment (Walter 1964; Carmon et al. 1976).

However, the vertex potential might also reflect neural activities important for initiating defensive motor responses to threat. This view receives compelling evidence from the fact that potentially threatening stimuli elicit both vertex potentials and defensive or protective movements (Graziano and Cooke 2006). These defensive movements are not only limited to reflexive and stereotyped subcortical motor responses (such as the flexion reflex; Sandrini et al. 2005), but also include cortically mediated, flexible and purposeful secondary reactions to a potential threat, occurring sometime after the vertex potential. Distinguishing between these interpretations of the functional significance of the vertex potential requires identifying specific criteria for defense behaviors. We suggest 2 candidate criteria: The spatial organization of the response (defensive behaviors should withdraw from a threatening stimulus, not approach it; Denny-Brown 1966; Vilensky and Gilman 1997), and the affective impact of the response (defensive behaviors should mitigate harm, either actual or potential).

In this study, we directly compare the salience and motoric interpretations of the vertex potential, by investigating the relationship between the amplitude of vertex potentials elicited by nociceptive-specific laser stimuli and defensive motor responses. Specifically, we compared responsestriggered by equally salient stimuli—which had different defensive values according to both the spatial organization criterion and the harm reduction criterion mentioned above. In a first experiment, we tested whether variability across trials in laser-evoked potential (LEP) amplitude predicts motor response time better when the motor response is defensive, or when it does not have a defensive value. In a second experiment, we tested whether the relation between LEP amplitude and response time differs between 2 movements that are kinematically identical, but have different harm reduction values.

## Materials and Methods

### Subjects

Forty-two healthy subjects participated in the study, which comprised 2 separate experiments. Twenty subjects (12 women) aged 20–37 years (mean ± SD = 27 ± 4.5 years) participated in Experiment 1, and 22 healthy subjects (8 women) aged 19–44 years (mean ± SD = 25 ± 6.3) participated in Experiment 2. All subjects provided written informed consent, and the experimental procedures were approved by the local ethics committee.

### Laser Stimulation (Experiments 1 and 2)

Noxious radiant heat pulses (4 ms duration) were generated by an infrared neodymium: yttrium-aluminum-perovskite (Nd : YAP) laser with a wavelength of 1.34 µm (Electronic Engineering, Florence, Italy). The laser beam was transmitted through an optic fiber, and its diameter at the target site set at approximately 6 mm (28 mm^2^). All laser stimuli were delivered on the dorsum of the right hand, and a He–Ne laser indicated the area to be stimulated. Before starting each experiment, we delivered a small number of low-energy laser pulses to the dorsum of the right hand to familiarize the subjects with the stimuli.

In Experiment 1, we used 2 stimulus intensities, individually adjusted to elicit the following average ratings of pricking pain: 3/10 (intensity “low”: 3.0 ± 0.5 J) and 6/10 (intensity “high”: 3.5 ± 0.5 J). We used a 0–10 rating scale, where 0 is “not painful at all” and 10 is “the worst pain imaginable” (Jensen et al. 1994).

In Experiment 2, we also used 2 stimulus intensities, individually adjusted to elicit the same 2 ratings of pricking pain: 3/10 (intensity “low”: 3.7 ± 0.4 J) and 6/10 (intensity “high”: 4.1 ± 0.4 J).

### Electrical Stimulation (Experiment 2)

Electrical stimuli were delivered using a surface bipolar electrode placed on the median nerve at the left wrist. They consisted of 500-ms long trains of electrical pulses at a frequency of 500 Hz. The duration of each pulse was 200 µs. The mean intensity of the stimulation was 6.6 ± 3.4 mA. This intensity was individually adjusted to elicit an aversive sensation of 6.5/10. We used a 0–10 scale, where 0 is “not aversive” and 10 is “the most aversive imaginable.”

### Experimental Design and Psychophysics

**Figure 1.**
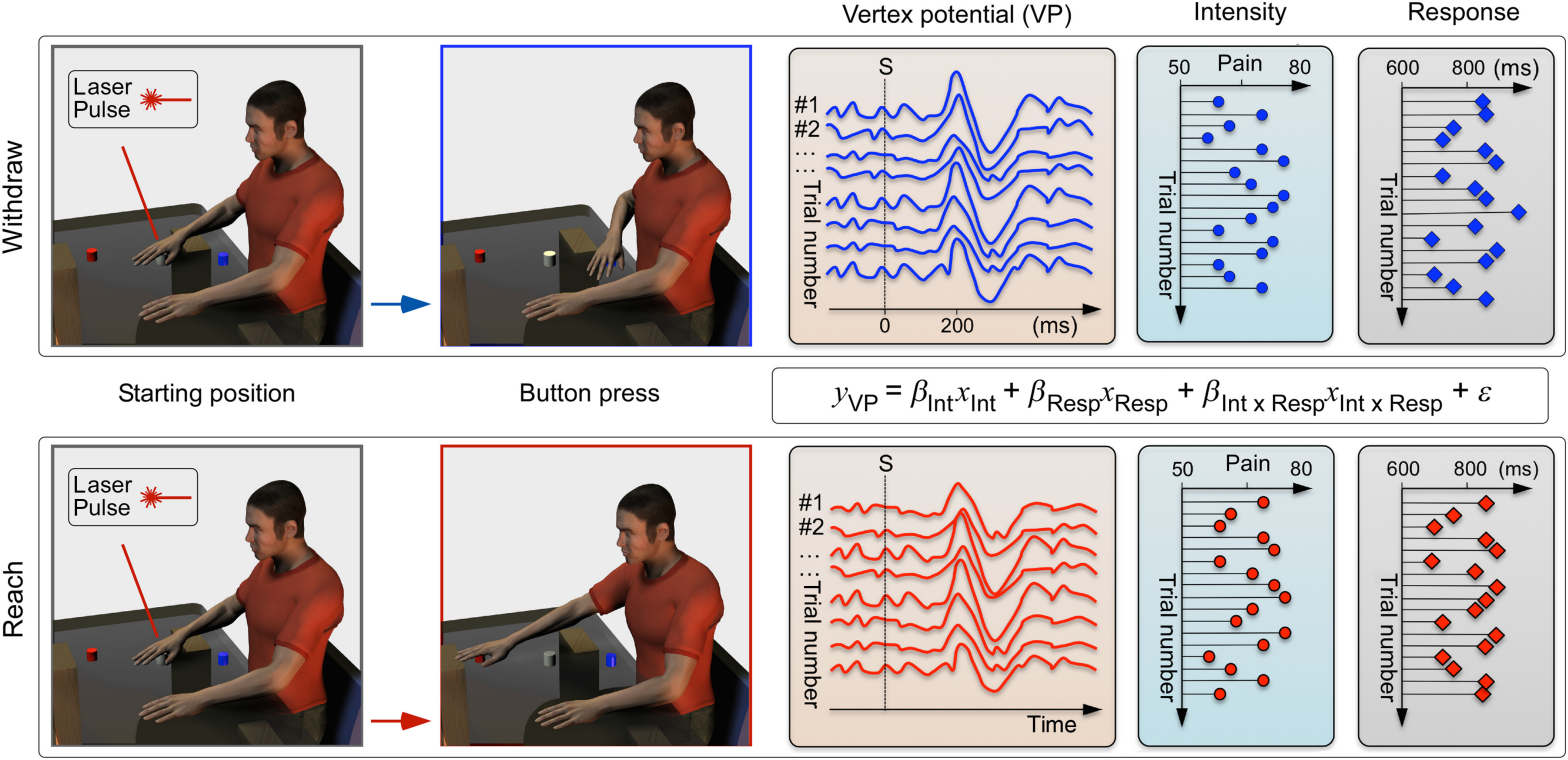
Design of Experiment 1. Participants performed a motor task in response to nociceptive laser stimulation of the right hand, while EEG was recorded. They were instructed to react as fast as possible when they felt the stimulus, by pressing a response button with the stimulated hand. In the “withdraw” condition, the button was placed approximately 10 cm from the body, at midline (i.e., ∼30 cm closer to the body with respect to the stimulated hand). In the “reach” condition, the button was placed ∼60 cm from the body, at midline (i.e., ∼20 cm further away from the hand stimulation position). Response times were defined as the time occurring between stimulus onset and the button press. Participants were instructed to provide a rating of the perceived pain intensity approximately 3 s after each trial. Trial-by-trial ratings, response times, and their interaction were used in a multiple linear regression that tested the relationship between these variables and vertex potential amplitude (see equation). These regression coefficients (*β*_Int_ and *β*_Resp_) were compared between conditions.

**Figure 2.**
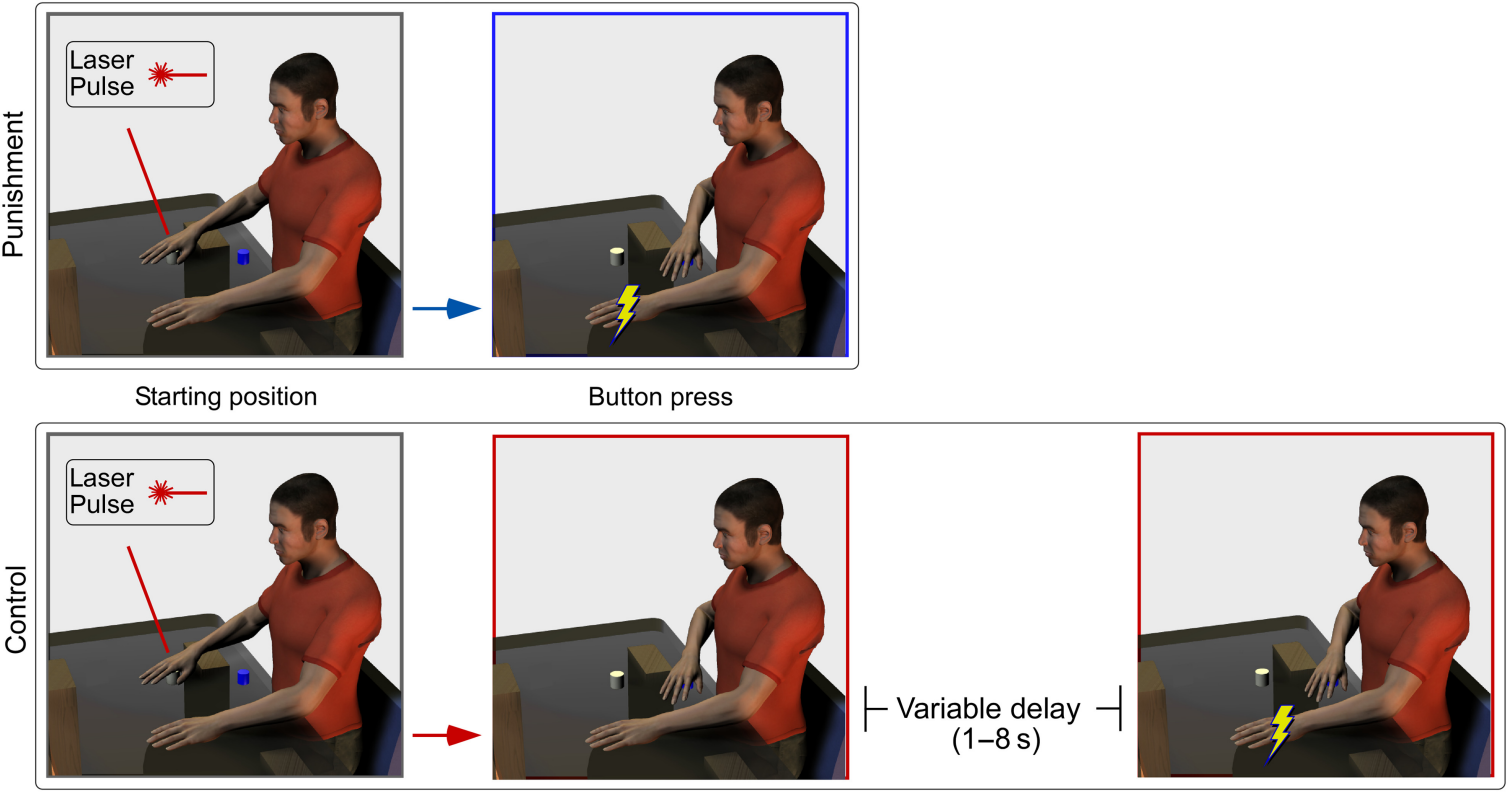
Design of Experiment 2. Participants performed a motor task in response to nociceptive laser stimulation of the right hand, while EEG was recorded. They were instructed to react as fast as possible when they felt the stimulus, by pressing a response button with the stimulated hand. The response button was placed approximately 10 cm from the body, at midline (i.e., ∼30 cm closer to the body with respect to the stimulated hand). In the “punishment” condition, the press of the response button immediately triggered an intense electrical stimulation of the left wrist. In the “control” condition, the electrical stimulation was not time-locked to the press of the response button; instead, it was delivered randomly, between 1 and 8 s after the button press. As in Experiment 1 (Fig. 1), participants were instructed to rate the intensity of the painful sensation elicited by the laser stimulus after each trial. Trial-by-trial ratings, response times, and their interaction were used in a multiple linear regression that tested the relationship between these variables and vertex potential amplitude.

In both experiments subjects sat comfortably, with their arms resting on a table placed in front of them, in a dimly lit, temperature-controlled room (Figs 1 and 2). Subjects were asked to focus on the laser stimuli, keep their eyes open and their gaze on a fixation cross (1.5 × 1.5 cm) placed at approximately 30 cm and 45° below eye level, about 20 cm to the left of the midline.

#### Experiment 1

The experiment consisted of a single recording session, divided into 4 blocks. In each block, we delivered 50 laser stimuli, 25 at low intensity and 25 at high intensity, on the right hand dorsum. The order of stimuli was pseudorandom, and the interstimulus interval ranged between 13 and 17 s (rectangular distribution). Subjects were required to keep their right hand approximately 40 cm from the body, at midline (Fig. 1). Subjects were instructed to react as fast as possible when they felt the laser stimulus, by pressing a response button (Fig. 1). In 2 of the 4 blocks (“reach” condition), the button was placed approximately 60 cm from the body, at midline (i.e., ∼20 cm further away from the hand stimulation position). In the other 2 blocks (“withdraw” condition), the button was placed approximately 10 cm from the body, at midline (i.e., ∼30 cm closer to the body with respect to the hand). The position of the 2 buttons was adjusted in each subject to achieve similar response times in the “reach” and “withdraw” conditions. This adjustment was based on a preliminary recording of response times to 20 stimuli (10 in the “reach” and 10 in the “withdraw” condition) delivered at an energy level halfway between the low and the high energies used in the main experiment. Therefore, to match reaction times in the “reach” and “withdraw” conditions, the distances between the response button and the hand stimulation position were different: In the “withdraw” condition, the distance was approximately 10 cm greater. The order of recording blocks was alternated within subject, and balanced across subjects. Response times, defined as the time occurring between stimulus onset and the button press, were recorded using an in-house script running under Matlab (version 7.5.0, Mathworks, Nantick, MA, USA). Subjects were instructed to provide a verbal rating of the subjective pain intensity approximately 3 s after each trial, and then return the hand to the stimulation position.

After the subject returned their hand to the stimulation position for the following trial, the laser beam was displaced to avoid stimulating the same spot and to prevent nociceptor fatigue or sensitization. Since variations in baseline skin temperature may affect pain perception (Tjolsen et al. 1988), we used an infrared thermometer to ensure that hand temperature remained constant across blocks.

#### Experiment 2

Experiment 2 also consisted of a single recording session, divided into 4 blocks. In each block, we delivered 50 laser stimuli, 25 at low intensity and 25 at high intensity, on the right hand dorsum. The order of stimuli was pseudorandom, and the interstimulus interval ranged between 9 and 13 s (rectangular distribution). Subjects were required to keep their hand on a switch placed approximately 40 cm from the body, at midline (Fig. 2). Subjects were instructed to react as fast as possible when they felt the laser stimulus, by releasing the switch and pressing a response button that, in contrast to Experiment 1, was always placed approximately 10 cm from the body, at midline (i.e., as in the “withdraw” condition of Experiment 1). In 2 of the 4 blocks, the press of the response button immediately triggered the electrical stimulation of the left wrist (“punishment” condition; Fig. 2). In the other 2 blocks (“control” condition), the electrical stimulation was not time-locked to the press of the response button; instead, it was delivered randomly, between 1 and 8 s after the button press, and always at least 4 s before the beginning of the next trial (Fig. 2). This ensured that there was no causal relationship between the button press and the punishment, thus maintaining the harm reduction impact of the withdrawal, while matching the total number of stimuli delivered in the 2 conditions. The order of recording blocks was balanced across subjects.

As in Experiment 1, subjects were instructed to provide a verbal rating of the subjective pain intensity elicited by the laser stimulus, approximately 3 s after each trial, and then return the hand to the stimulation position.

### EEG Recording

The EEG was recorded using 32 Ag–AgCl electrodes placed on the scalp according to the International 10-20 system and referenced to the nose. Electrode impedances were kept below 5 kΩ. The electrooculogram (EOG) was recorded from 2 surface electrodes, one placed over the right lower eyelid and the other placed lateral to the outer canthus of the right eye. Signals were amplified and digitized at a sampling rate of 1024 Hz (SD32; Micromed, Treviso, Italy).

### Behavioral Data Analysis

In both Experiments, response times and subjective pain intensities were compared between experimental conditions in each subject, using paired *t*-tests. Statistical threshold was set at *P* = 0.05.

### EEG Analysis

#### Preprocessing

EEG data were preprocessed and analyzed using Letswave4 (www.nocions.org/letswave) (Mouraux and Iannetti 2008) and EEGLAB (Delorme and Makeig 2004). Continuous EEG data were segmented into epochs using a time window ranging from 0.5 s before to 1 s after stimulus onset (total epoch duration: 1.5 s), and bandpass-filtered from 1 to 30 Hz. Each epoch was baseline-corrected using the interval −0.5 to 0 s as a reference. Artifacts due to eye blinks or eye movements were removed using a validated method based on independent component analysis (Jung et al. 2000). In all data sets, independent components related to eye movements had a large EOG channel contribution and a frontal scalp distribution. Finally, epochs with amplitude values greater than ±100 µV (i.e., likely contaminated by artifacts) were excluded from the analysis.

#### Standard Averaging Analysis

Epochs belonging to the same experimental condition (“reach” and “withdraw” for Experiment 1; “punishment” and “control” for Experiment 2) were averaged time-locked to stimulus onset, for each subject. The 3 main LEP peaks (N1, N2, and P2) were identified in each average waveform, as follows. The N2 wave was defined as the most negative deflection after stimulus onset, at Cz. The P2 wave was defined as the most positive deflection after stimulus onset, at Cz. N1 was identified at Cc-Fz (Hu et al. 2010), and defined as the negative deflection preceding the N2 wave, which appears as a positive deflection in this montage. To ensure that the LEPs elicited in different experimental conditions were not different, peak amplitudes of the N1, N2, and P2 waves were compared using paired *t*-tests. The threshold for statistical significance was set at *P* = 0.05.

#### Single-Trial Analysis

To determine whether there was a relationship between LEP amplitude at Cz and the response time while controlling for pain-related variability, we used a multiple linear regression method (Mayhew et al. 2006). We focused our analysis on Cz because it is the electrode where the negative and positive waves composing the vertex potential are maximal. Briefly, we calculated a regression coefficient (*β*-value) for pain intensity (*β*_Int_), response time (*β*_Resp_), and their interaction (*β*_Int__×__Resp_) for each condition. A detailed description of this procedure can be found in the Supplementary Methods.

Given the previously identified relationship between response time and pain intensity, we evaluated the estimability of these predictors using the Belsley's Collinearity test (Belsley 1991), as implemented in the “collintest” function, in Matlab. Importantly, all 3 predictors were never found to exceed a very stringent tolerance (i.e., a condition index of >5 and a variance decomposition proportion of >0.5) in every single subject, for both experiments. This result indicates that all 3 variables are suitable to be included as regressors in a multiple linear regression model.

In each Experiment, we tested for differences between experimental conditions, for both *β*_Int_ and *β*_Resp_ (Experiment 1: “withdraw” vs. “reach”; Experiment 2: “punishment” vs. “control”), using point-by-point paired *t*-tests combined with nonparametric permutation testing (Maris and Oostenveld 2007). The threshold for statistical significance was set at *P* = 0.05. To account for multiple comparisons, significant time points (*P* < 0.05) were categorized in clusters based on their temporal adjacency (cluster-level statistical analysis). Only clusters composed of >30 adjacent significant timepoints were considered, and only the largest cluster was selected to control for false-positive observations. The cluster-level statistics ($\sum_{T}$) was defined by calculating the sum of the *t-*values of all time points within a cluster. The *β*-values were then randomly permutated 5000 times. In each *m*_th_ permutation, the same paired-sample *t*-test was performed on the randomly permutated *β*-values, which yielded a cluster-level statistics $\sum_{T}^{*}(m)$. Permutation distributions $D(\sum_{T})$ of the cluster-level *t*-statistics were obtained from all $\sum_{T}^{*}(m)$, and the two-tailed *P*-value *P_T_* was obtained by locating the observed $\sum_{T}$ under the permutation distribution $D(\sum_{T})$for each cluster.

**Figure 3.**
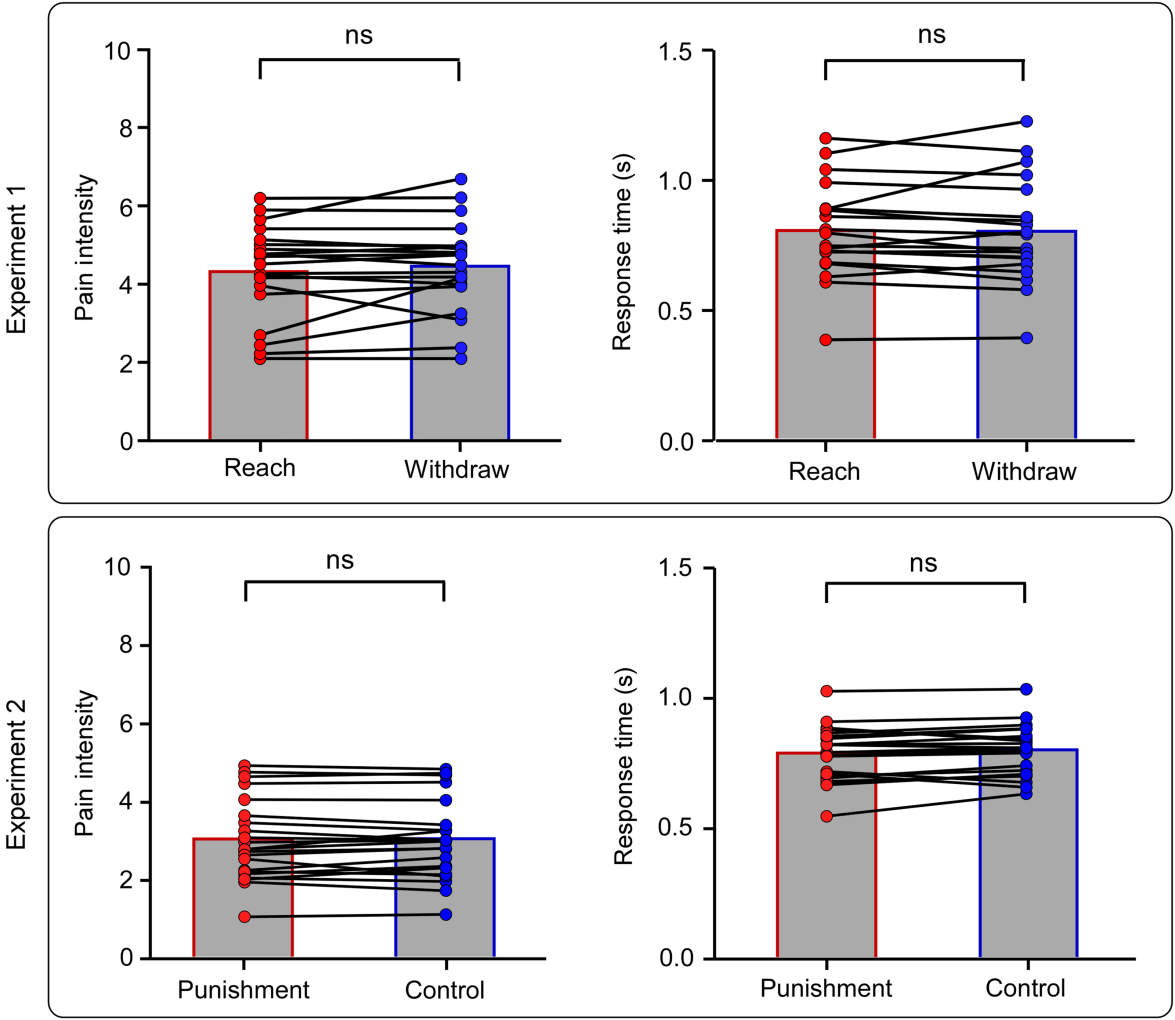
Behavioral results. Single-subject pain intensity ratings and response times for the experimental conditions of Experiments 1 (*n* = 20) and 2 (*n* = 22). Columns define group averages. ns: non-significant. Reaction time data are presented in Supplementary Figure 1.

**Figure 4.**
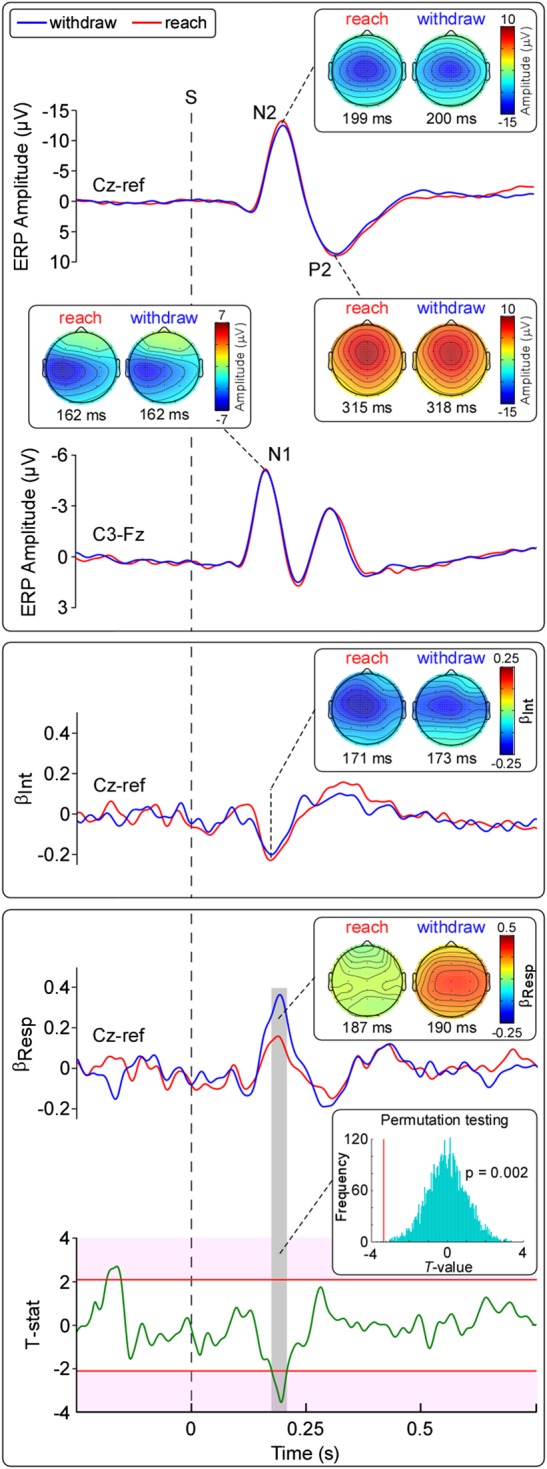
Experiment 1. LEP waveforms and multiple linear regression results (*n* = 20). Top panel: Group-level LEPs and scalp topographies. Note the similarity of responses in the “reach” and “withdraw” conditions. Middle panel: Time course of coefficients of the intensity regressor (*β*_Int_) and peak scalp topographies. Note the similarity of the *β*_Int_ waveforms and scalp topographies in the 2 conditions. Bottom panel: Time course of coefficients of the response time regressors (*β*_Resp_) and peak scalp topographies. The lower waveform shows the *T*-values of the comparison between “reach” and “withdraw” *β*_Resp_. Significance threshold (critical value, *T* = 2.10) is shown by the red line. The inset shows the significant difference, using permutation testing (5000 iterations). Note that the only significant difference between the 2 *β*_Resp_ (highlighted in gray) falls in the N2 time window. Note also the dissimilar scalp topography of the *β*_Resp_ in the 2 conditions. Reaction time data are presented in Supplementary Figure 2.

**Figure 5.**
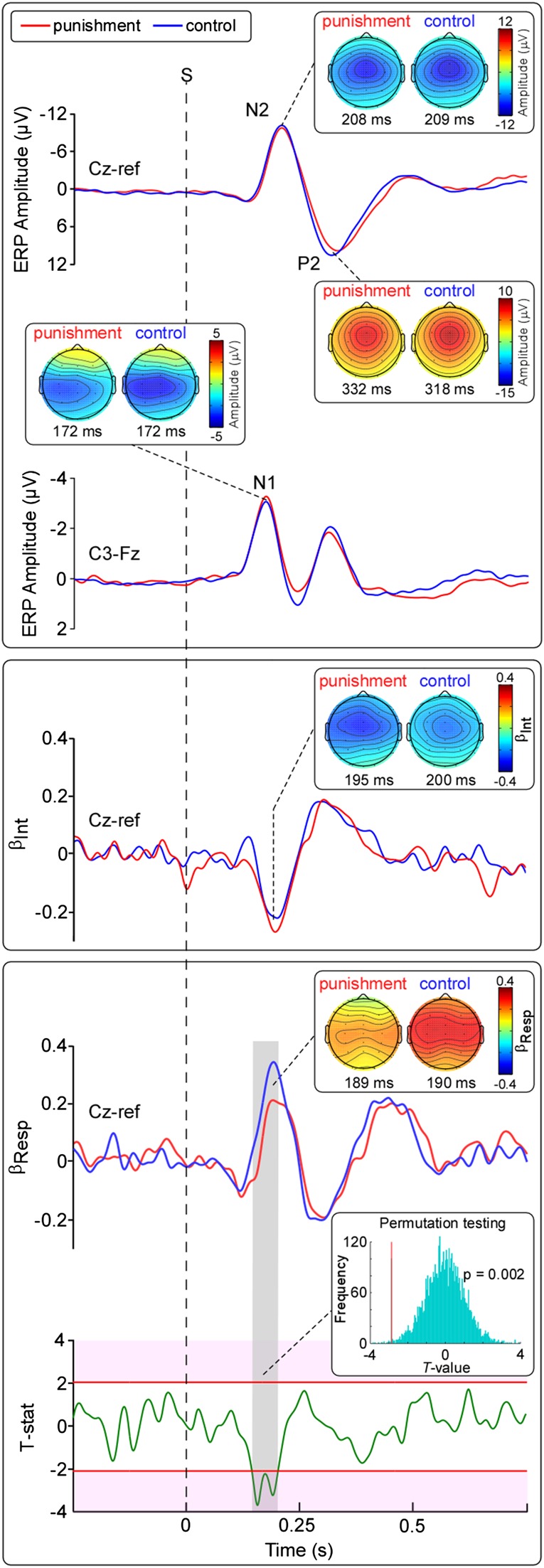
Experiment 2. LEP waveforms and multiple linear regression results (*n* = 22). Top panel: Group-level LEPs and scalp topographies. Note the similarity of responses in the “punishment” and “control” conditions. Middle panel: Time course of coefficients of the intensity regressor (*β*_Int_) and peak scalp topographies. Note the similarity of the *β*_Int_ waveforms and scalp topographies in the 2 conditions. Bottom panel: Time course of coefficients of the response time regressors (*β*_Resp_) and peak scalp topographies. The lower waveform shows the *T*-values of the comparison between “punishment” and “control” *β*_Resp_. Significance threshold (critical value, *T* = 2.08) is shown by the red lines. The inset shows the significant difference, using permutation testing (5000 iterations). Note that the only significant difference between the 2 *β*_Resp_ (highlighted in gray) falls in the N1 and N2 time windows. Note that the scalp topographies of the *β*_Resp_ in the “control” and “punishment” conditions are similar, despite the difference in amplitudes. Reaction time data are presented in Supplementary Figure 3.

## Results

### Experiment 1

#### Behavioral Results

In all subjects, laser stimuli elicited a clear sensation of pinprick pain, related to the activation of Aδ fibers (Bromm and Treede 1984). Pain ratings and response times for each condition are shown in Figure 3. As expected, both pain ratings (reach: 4.3 ± 1.2; withdraw: 4.5 ± 1.2; *P* = 0.29) and response times (reach: 805 ± 182 ms; withdraw: 802 ± 199 ms; *P* = 0.84) were matched between conditions. Similar results were obtained when examining reaction times (Supplementary Results).

#### LEP Waveforms

Grand average waveforms of the LEPs obtained in the 2 experimental conditions are shown in Figure 4. The amplitudes of the main LEP waves were not different in the reach and withdraw conditions (N1: *P* = 0.81; N2: *P* = 0.072; P2: *P* = 0.91). The corresponding scalp topographies in the reach and withdraw conditions were also remarkably similar. The negative (N2) and positive (P2) waves of the vertex potential were maximal at the scalp vertex (electrode Cz). The N2 wave extended bilaterally toward temporal regions, whereas the P2 was more centrally distributed (Fig. 4).

#### Single-Trial Analysis

Figure 4 shows the time course of the coefficients of the intensity and response time regressors (*β*_Int_ and *β*_Resp_, respectively), for the reach and withdraw conditions. These coefficients reflect whether the trial-by-trial variability of the LEP amplitude was able to predict the subjective pain intensity (*β*_Int_) and the response time (*β*_Resp_). The *β*_Int_ for reach and withdraw conditions were not significantly different. Indeed, the *β*_Int_ showed a significant negative relationship between the LEP amplitude and intensity ratings in the N1 and N2 time windows (*P* < 0.05), both in the withdraw and in the reach conditions. Similarly, the scalp topographies of the peak value of *β*_Int_ were not different: They were maximal at the midline (Cz) and at the central electrodes contralateral to the stimulated hand (C3), and extended bilaterally toward the temporal regions.

The *β*_Resp_ showed a significant positive relationship between the LEP amplitude and response times in the N1 and N2 time windows (*P* < 0.05), and a negative relationship in the P2 time window (*P* < 0.05). Crucially, the *β*_Resp_ in the reach and withdraw conditions were significantly different—the trial-by-trial variability of LEP amplitude better predicted response times in the withdraw condition than in the reach condition (*P* < 0.05; Fig. 4, lower panel). This difference was present in the time window corresponding to the latency of the N2 wave (176–207 ms, *P* < 0.05), but not in the time windows corresponding to the latency of the N1 and P2 waves. Similar results were obtained when examining reaction times (Supplementary Results).

### Experiment 2

#### Behavioral Results

In all subjects, laser stimuli elicited a clear sensation of pinprick pain. Pain ratings and response times for each condition are shown in Figure 3. As expected, both pain ratings (punishment: 3.0 ± 1.1; control: 3.0 ± 1.0; *P* = 0.82) and response times (799 ± 98 ms, control: 785 ± 105, *P* = 0.10) were matched between conditions. Similar results were obtained when examining reaction times (Supplementary Results).

#### LEP Waveforms

Grand average waveforms of the LEPs obtained in the 2 experimental conditions are shown in Figure 5. The amplitudes of the main LEP waves were not different in the punishment and control conditions (N1: *P* = 0.27; N2: *P* = 0.63; P2: *P* = 0.93). The corresponding scalp topographies in the punishment and control conditions were also remarkably similar. The N2 and P2 waves were maximal at the scalp vertex (electrode Cz). The N2 extended bilaterally toward temporal regions, whereas the P2 was more centrally distributed (Fig. 5).

#### Single-Trial Analysis

Figure 5 shows the time course of the coefficients of the intensity and response time regressors (*β*_Int_ and *β*_Resp_, respectively) for the punishment and control conditions. The *β*_Int_ for punishment and control conditions were not significantly different. Indeed, the *β*_Int_ showed a significant negative relationship between the LEP amplitude and intensity ratings in the N1 and N2 time windows (*P* < 0.05). There was also a positive relationship between the *β*_Int_ in the P2 time window (*P* < 0.05). The scalp topographies of the peak value of *β*_Int_ were similar: They were maximal at the midline (Cz) and at the central electrodes contralateral to the stimulated hand (C3), and extended bilaterally toward the temporal regions.

The *β*_Resp_ showed a significant positive relationship between the LEP amplitude and response times in the N1 and N2 time windows (*P* < 0.05), and a negative relationship in the P2 time window (*P* < 0.05). The scalp topographies of the peak values of the *β*_Resp_ were similar, although their magnitude was clearly different (Fig. 5). Indeed, the trial-by-trial variability of LEP amplitude better predicted response times in the control condition than in the punishment condition (corrected *P* < 0.05; Fig. 5, lower panel). This difference was only present in a time window corresponding to the latency of the N1 and N2 wave (146–202 ms, *P* < 0.05), but not in the time window corresponding to the latency of the P2 wave. Similar results were obtained when examining reaction times (Supplementary Results).

## Discussion

We have provided answers to critical questions regarding the functional significance of the vertex potentials elicited by nociceptive laser stimuli. Our study is the first, to our knowledge, to show that the laser-evoked negative vertex wave (N2) is not simply a by-product of detecting salient stimuli. Rather, it includes neural activities important for the initiation and the execution of cortically mediated defensive actions. This conclusion was based on analyzing 2 key attributes that distinguish defensive actions from other types of actions: Spatial organization for withdrawal (Experiment 1), and association with harm reduction (Experiment 2). Based on these criteria, we conclude that the laser-evoked vertex potential is important for “defensive agency.”

Our experiments yielded 2 main findings. First, the N2 wave better predicts how rapidly individuals perform a defensive movement compared with a non-defensive movement (Fig. 4). Importantly, even the reaction time (i.e., onset of the movement) is better predicted by the N2 amplitude in a defensive context (Supplementary Fig. 2). Therefore, the strength of the relationship between N2 amplitude and movement speed is dictated by the extent to which the task includes a functional response to threat. This threat-specificity is further demonstrated by our second finding: When a punishment consisting of an intense and aversive electrical shock is triggered by the withdrawal movement, thus strongly reducing its defensive value, the relation between the N2 wave and the speed of the subsequent movement is reduced (Fig. 5). These results provide, for the first time, important insights regarding the function of the laser-evoked vertex potential. In particular, they suggest that some of the neural activities reflected by the vertex potential play a significant integrative role, bridging the detection of threat to appropriate defensive actions.

### N2 Wave Amplitude Predicts Perceived Intensity and Reaction Times

The vertex potential, first described over 60 years ago, is the largest synchronization of neural activity observed in the human brain of a healthy, awake individual (Bancaud et al. 1953; Walter 1964). Despite intense study of the rules defining the criteria by which a sensory event elicits a vertex potential, the function of this signal has yet to be understood. Specifically, the nervous system is tuned to detect salient changes in the sensory environment, such as modality changes and increases in stimulus intensity (Valentini et al. 2011; Ronga et al. 2013). Nonetheless, the understanding of these rules does not provide information about the mechanisms through which the vertex potential affects behavior.

Here, we showed that the laser-evoked N2 vertex wave predicts both the reaction times and the response times of a defensive motor action (Figs 4 and 5; Supplementary Figs 2 and 3). Three relevant correlations have been identified in previous studies. First, there is a relationship between the trial-by-trial variation in the vertex potential amplitude and reaction times (Donchin and Lindsley 1966; Wilkinson and Morlock 1967; Wastell and Kleinman 1980). Second, there is a well-established relation between reaction times and perceived stimulus intensity, with higher intensities being related to shorter reaction times (e.g., Cattell 1886; Piéron 1914; Angel 1973; Arendt-Nielsen and Bjerring 1988). Third, perceived stimulus intensity is proportional to vertex potential amplitude (e.g., Diamond 1964; Giblin 1964; Iannetti et al. 2008; Hu et al. 2014). Importantly, previous studies investigating the relationship between reaction times and vertex potential amplitude did not attempt to separate these various inter-related components, nor did they account for the valence of the movement outcome. In particular, no previous study, to our knowledge, has investigated the relationship between vertex potential amplitude and movement parameters, after controlling for the effects of perceived intensity. We used a multiple regression approach to account for these interactions. By controlling for (1) perceived stimulus intensity and (2) the interaction between perceived intensity and response time, we were able to identify the unique portions of trial-by-trial variance that can be explained by perceived pain intensity (*β*_Int_) and by response time (*β*_Resp_). Furthermore, by manipulating the defensive meaning of the movement, we were able to test whether the laser-evoked vertex potential encodes the kinematics of the movement or its behavioral outcome.

### The N2 Wave Is Specifically Tuned to Parameters of Threat-Related Movements

We hypothesized that the vertex potential is related to the execution of defensive motor actions. Therefore, we investigated the trial-by-trial relationship between LEP amplitude and response time of 3 types of movements with different defensive values.

In Experiment 1, we explored 2 different movements: A withdrawal and a reach. Although both movements are clearly goal-directed (i.e., the subject must press a button at a target location), only the withdrawal movement closely resembles natural movements related to the defense of the body (Graziano 2008). In contrast, the reaching movement involves approach to an external object—a direction of movement that characterizes instrumental actions with the external world, as opposed to withdrawal from it (Jeannerod 1988). In other words, withdrawal is a spatially organized motor response, aiming to defend the body, and is elicited by salient stimuli that represent potential threats (Graziano 2006). Such defensive movements take into account the location and trajectory of proximal objects, the body region that is threatened, and various other stimulus properties (Cooke and Graziano 2003). There is strong, causal evidence that the spatial and kinematic patterns of such movements are represented in the frontal lobes, and particularly the premotor cortex (Cooke and Graziano 2004). We found that the speed of a goal-oriented movement is predicted by the amplitude of the preceding N2 wave, but that this relation is significantly stronger for withdrawal than for reaching movements (Fig. 5).

We took care that both response times and subjective pain intensities were matched across conditions (Fig. 3). Given that we sought to match response times, the trajectory distance to the reach and withdraw targets was different. Thus, *Experiment 1* involved comparing withdrawal and reaching movements with rather different kinematics. To address this limitation, in *Experiment 2*, we had subjects perform the exact same movement, but we altered the affective value of the movement. Specifically, in one condition, we paired the movement with a punishment (Fig. 2). By this means, we introduced a difference between conditions in the defensive value of a single movement. We found that, in the punishment condition, the relationship between the N2 wave and the withdrawal was reduced, relative to the control condition (Fig. 5).

Interestingly, the scalp distribution of the relation between N2 wave amplitude and response time differed between defensive movement and control movement (Experiment 1, Fig. 4). The scalp maps for the withdrawal condition showed a single maximum at the vertex, whereas that for the reach condition showed 2 symmetrical maxima at the centro-parietal electrodes. This observation suggests that the execution of defensive actions might be related to the activity of a specific set of neural generators contributing to the N2 wave of the vertex potential. Whether the difference in *β*_Resp_ between defensive and non-defensive actions reflects neural activities directly implicated in planning and initiating defensive movements, or whether it somehow facilitates downstream neural activities that plan and initiate those movements, is an open question.

Notably, the interpretation that it is the defensive context that modulated the ability of the N2 wave amplitude to predict a subsequent movement is further supported by the finding that the N2 wave amplitude predicts not only the response time (Figs 4 and 5), but also the reaction time of the movement (Supplementary Figs 2 and 3). In our study, the reaction time represents the time it takes the subject to release the switch to perform the required movement—i.e., the movement has not yet occurred. Therefore, the observation that both reaction and response times are predicted by the trial-by-trial variability of the N2 amplitude suggests that a subset of the neural activities reflected in the vertex potential is related to the motor planning of defensive actions.

### A Putative Model: Defensive Motor Actions Are Driven by Mid-Cingulate Cortex–Premotor Connections

The observed relationship between the N2 wave amplitude and the response time of a subsequent defensive movement suggests that some of the neural generators of the vertex potential might overlap with neural circuitry mediating the planning of defensive motor actions. Among the generators contributing to the N2 wave is the mid-cingulate cortex [MCC, based on the nomenclature proposed by Vogt (2009); previously labeled as ACC, e.g., Rios et al. 1999; Garcia-Larrea et al. 2003]. It has been proposed that the MCC serves as a hub between affective processing and motor planning (Vogt and Sikes 2009; Shackman et al. 2011; Morrison et al. 2013; Perini et al. 2013). Support for the concept of an “affective premotor cingulate” comes from several converging lines of evidence. For example, escape-related neurons have been found in monkeys, in a region analogous to the human MCC (Iwata et al. 2005), and surgical ablation of the MCC increases the escape threshold to nociceptive stimulation (Pastoriza et al. 1996; Donahue et al. 2001). Furthermore, the MCC is the only cingulate region to receive input from the spinothalamic tract (Dum et al. 2009), and has direct projections to cortical motor regions and motoneurons in the brainstem and the spinal cord (Dum and Strick 1992). Furthermore, the MCC is strongly connected with the premotor cortex (Beckmann et al. 2009), a region that has been shown to initiate defensive motor actions (Cooke and Graziano 2003). These connections might provide a neural substrate for the stronger *β*_Resp_ in conditions entailing the execution of a defensive movement.

It is important to note that, given the intrinsic limitations of source analysis of scalp EEG signals (Mouraux and Iannetti 2008), it is difficult to reliably identify the source of a widespread wave such as the N2. Therefore, we cannot rule out the contribution of neural activity arising from other midline structures such as the supplementary motor area, the pre-supplementary motor areas, and other subregions of the cingulate cortex.

In conclusion, we investigated the relationship between the vertex potential amplitude and defensive motor actions in a simple manual response task. Our results demonstrate that the laser-evoked N2 vertex wave reflects neural activities important for planning and initiating defensive actions.

## Supplementary Material

Supplementary material can be found at: http://www.cercor.oxfordjournals.org/.

## Funding

G.D.I. acknowledges the support of The Wellcome Trust. M.M. has a Fellowship Award from the Canadian Institutes of Health Research and the IASP Scan|Design International Trainee Fellowship. P.H. is supported by ERC Advanced Grant HUMVOL, and by an ESRC Professorial Fellowship. Funding to pay the Open Access publication charges for this article was provided by The Wellcome Trust.

## Supplementary Material

Supplementary Data
