# Supplementary material for: Laser-Evoked Vertex Potentials Predict Defensive Motor Actions
Source: Cereb Cortex. 2015 Aug 6;25(12):4789–98. doi: 10.1093/cercor/bhv149 (PMC4635919; doi:10.1093/cercor/bhv149)
Supplement: Supplementary Data [file supp_25_12_4789__index.html]

Laser-Evoked Vertex Potentials Predict Defensive Motor Actions — Supplementary Data 

# Laser-Evoked Vertex Potentials Predict Defensive Motor Actions

## Supplementary Data

Supplementary Data

- Supplementary Data - Docx file
