# Supplementary material for: Laser-Evoked Vertex Potentials Predict Defensive Motor Actions
Source: Cereb Cortex. 2015 Aug 6;25(12):4789–98. doi: 10.1093/cercor/bhv149 (PMC4635919; doi:10.1093/cercor/bhv149)

**Supplementary Material for:**

Laser-evoked vertex potentials predict defensive motor actions

M Moayedi^1^, M Liang^1^, AL Sim^1^, L Hu^1,2^, P Haggard^3^, GD Iannetti^1^

*^1^Department of Neuroscience, Physiology and Pharmacology, University College London, London WC1E 6BT, UK; ^2^Key Laboratory of Cognition and Personality (Ministry of Education) and School of Psychology, Southwest University, Chongqing, 400715, China; ^3^Institute of Cognitive Neuroscience & Department of Psychology, University College London, London, WC1N 3AR, UK*

**Supplementary Methods**

*Reaction times.* We collected reaction times (i.e., onset of movement) in 12 out of 20 subjects participating in Experiment 1 (mean ±SD = 26 ±3.9 years; range: 22-34 years), and in all subjects participating in Experiment 2. In these subjects the hand was resting on a switch in the stimulation position, (approximately 40 cm from the body, at midline) and the movement onset was defined as the moment when the button was released to initiate the movement. Subjects were instructed to react as fast as possible when they felt the laser stimulus, by releasing the switch and pressing the response button. Therefore, in these subjects we recorded not only the response time (i.e. the time needed to press the response button), but also the reaction time.

*Behavioral data analysis.* In both Experiment 1 and 2, reaction times were compared between experimental conditions in each subject, using paired t-tests. Statistical threshold was set at p=0.05.

*Single-trial analysis.* To determine whether there was a relationship between LEP amplitude at Cz and the reaction time while controlling for input variability, we used a multiple linear regression method (see below, and Mayhew et al., 2006). Briefly, we calculated, for each condition, a regression coefficient (β value) for pain intensity (β_Int_), reaction time (β_RT_), and their interaction (β_Int x RT_).

Given the previously identified relationship between reaction time and pain intensity, we evaluated the estimability of these predictors using the Belsley’s Collinearity test (Belsley, 1991), as implemented in the *collintest* function in Matlab. Importantly, all three predictors were never found to exceed a very stringent tolerance (i.e., a condition index >5 and a variance decomposition proportion >0.5) in every single subject, for both experiments. This result means that all three variables are suitable to be included as regressors in a multiple linear regression model.

In each Experiment, we tested for differences between experimental conditions, for both β_Int_ and β_RT_ (Experiment 1: ‘withdraw’ vs ‘reach’; Experiment 2: ‘punishment’ vs ‘control’), using point-by-point paired t-tests. The threshold for statistical significance was set at p=0.05.

*Multiple linear regression*. The model was applied to preprocessed EEG data, and can be written as:

$$y_{LEP}=+\beta_{Int}x_{Int}{+\beta}_{Resp}x_{Resp}+\beta_{Int \times Resp}x_{Int \times Resp}+\varepsilon$$


where y_LEP_ is a m × n matrix containing m single-trials EEG epochs and n samples of each epoch (the LEP waveform from -500 to 1000 ms after stimulus onset). X_i_ are the regressors, namely trial-by-trial pain intensity ratings (x_int_) and trial-by-trial response times (x_Resp_), and their interaction (x_int x Resp_). β_i_ are coefficients that weight the fit of X_i_ to the data y. ɛ is the residual error of the fitted model. The coefficients β_i_ for each condition were calculated by multiple linear regression.

A similar equation was used to assess the relationship between reaction time and LEP amplitude, as follows:

$$y_{LEP}=+\beta_{Int}x_{Int}{+\beta}_{RT}x_{RT}+\beta_{Int \times RT}x_{Int \times RT}+\varepsilon$$

**Supplementary Results**

We collected reaction times (i.e., onset of movement) in 12 out of 20 subjects participating in Experiment 1, and in all subjects participating in Experiment 2. Reaction time results are presented herein.

*Behavioural results.* Pain ratings and reaction times for each condition are shown in Figure S1. In *Experiment 1*, pain ratings (reach: 4.6 ±1.3, withdraw: 4.7 ±1.3, p=0.84) and reaction times (reach: 500 ±121 ms, withdraw: 498 ±118 ms; p=0.82) were matched between conditions. In *Experiment 2*, pain ratings (punishment: 3.0 ±1.0, control: 3.0 ±1.0, p=0.83) and reaction times (punishment: 532 ±69 ms, control: 542 ±54 ms; p=0.24) were also matched between conditions.

*Experiment 1*

*LEP waveforms.* Grand average LEP waveforms obtained in the two conditions are shown in Figure S2. The amplitudes of the main LEP waves were not different in the reach and withdraw conditions (N2: p=0.61; P2: p=0.92). The corresponding scalp topographies in the reach and withdraw conditions were also remarkably similar. The negative (N2) and positive (P2) waves were maximal at the scalp vertex (electrode Cz). The topography of the N2 wave extended bilaterally toward temporal regions whereas the topography of the P2 was more centrally distributed (Figure S2).

*Single-trial analysis.* Figure S2 shows the time-course of the coefficients of the intensity and reaction time regressors (β_Int_ and β_RT_, respectively) for the reach and withdraw conditions. These coefficients reflect whether the trial-by-trial variability of the LEP amplitude predicts the subjective pain intensity (β_Int_) and the reaction time (β_RT_). The β_Int_ for reach and withdraw conditions were not significantly different. Indeed, the β_Int_ showed a significant negative relationship between LEP amplitude and intensity ratings in the N1 and N2 time windows (p<0.05), both in the withdraw and the reach conditions. Similarly, the scalp topographies of the peak value of β_Int_ were not different: they were maximal at the central electrodes, and extended bilaterally towards temporal regions.

The β_RT_ showed a significant positive relationship between the LEP amplitude and reaction times in the N1 and N2 time windows (p<0.05), and a negative relationship in the P2 time window (p<0.05). Crucially, the β_RT_ for reach and withdraw were significantly different – the trial-by-trial variability of LEP amplitude better predicted reaction times in the withdraw condition than in the reach condition (p<0.05; Figure S2, lower panel). This difference was present in a time window corresponding to the latency of the N2 wave (190-212 ms, p<0.05), but not in the time window corresponding to the latency of the N1 and P2 waves.

*Experiment 2*

*LEP waveforms.* Grand average LEP waveforms obtained in the two experimental conditions are shown in Figure 5 and Figure S3. The amplitudes of the main LEP waves were not different in the punishment and control conditions (N1: p=0.27; N2: p=0.63; P2: p=0.93). The corresponding scalp topographies in the punishment and control conditions were also remarkably similar. The N2 and P2 waves were maximal at the scalp vertex (electrode Cz). The topography of the N2 wave extended bilaterally toward temporal regions whereas the topography of the P2 wave was more centrally distributed (Figure 5 and Figure S3).

*Single-trial analysis.* Figure S3 shows the time-course of the coefficients of the intensity and reaction time regressors (β_Int_ and β_RT_, respectively) for the punishment and control conditions. The β_Int_ for punishment and control conditions were not significantly different. The β_Int_ showed a significant negative relationship between the LEP amplitude and intensity ratings in the N1 and N2 time windows (p<0.05). The scalp topographies of the peak value of β_Int_ were similar: they were maximal at the central electrodes, and extended bilaterally towards temporal regions.

The β_RT_ showed a significant positive relationship between the LEP amplitude and reaction times in the N2 time windows (p<0.05), and a negative relationship in the P2 time window (p<0.05). The scalp topographies of the peak values of the β_RT_ were similar, although their magnitude was clearly different (Figure S3). Indeed, the trial-by-trial variability of LEP amplitude better predicted reaction times in the control condition than in the punishment condition (p<0.05; Figure S3, lower panel). This difference was only present in a time window corresponding to the latency of the N1 and N2 waves (146-202 ms, p<0.05), but not in the time window corresponding to the latency of the P2 wave.

**Supplementary References**

Belsley, D.A. (1991). A Guide to using the collinearity diagnostics. Computer Science in Economics and Management *4*, 33-50.

Mayhew, S.D., Iannetti, G.D., Woolrich, M.W., and Wise, R.G. (2006). Automated single-trial measurement of amplitude and latency of laser-evoked potentials (LEPs) using multiple linear regression. Clin Neurophysiol *117*, 1331-1344.

**Supplementary Figures Legends**

**Figure S1. Behavioural results of reaction time datasets.** Single-subject pain intensity ratings and reaction times for the experimental conditions of Experiments 1 (n=12/20) and 2 (n=22/22). Columns define group averages. n.s. = non significant.


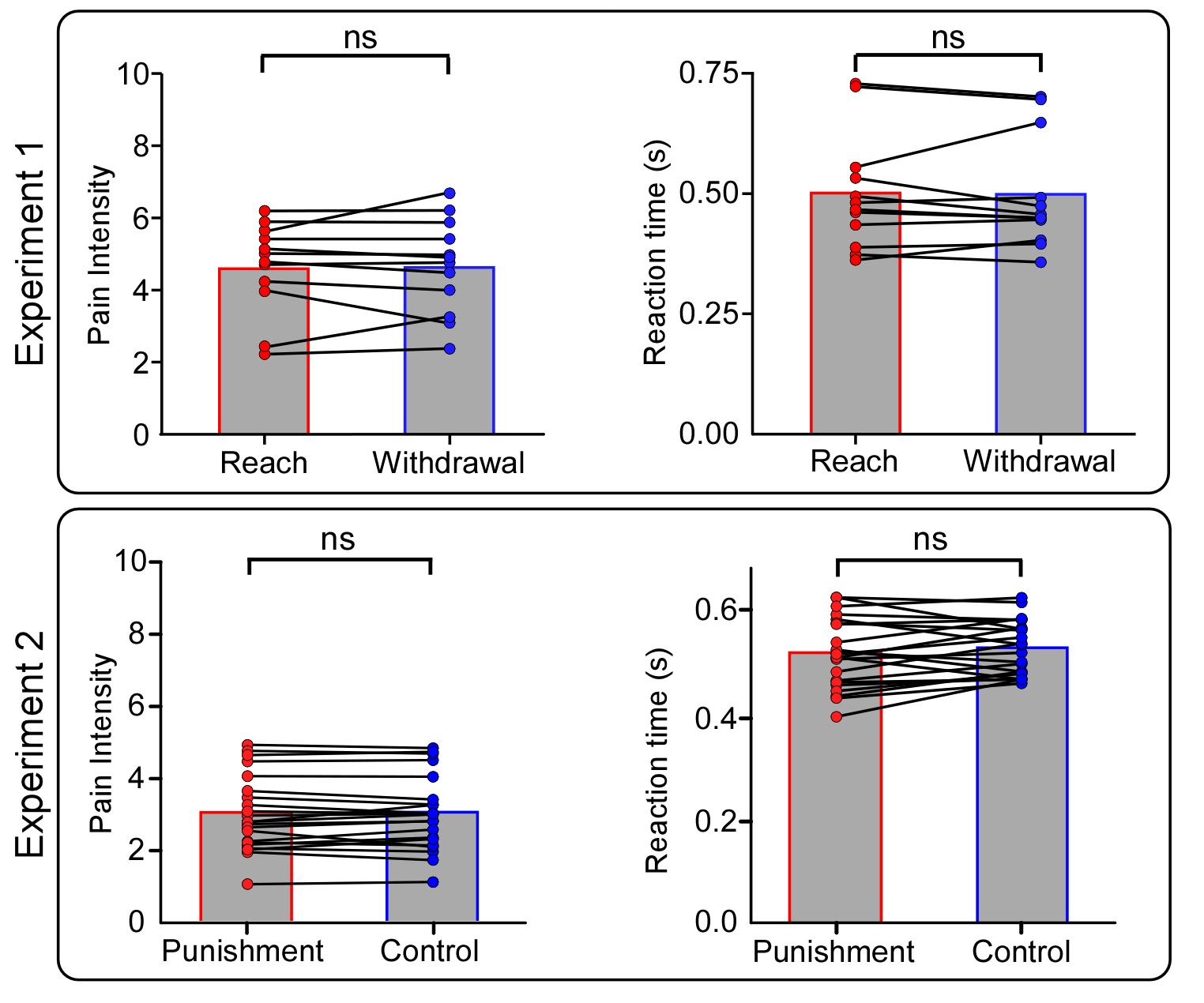


**Figure S2. Experiment 1. LEP waveforms and multiple linear regression results of reaction time dataset (n=12/20).** Top panel: Group-level LEPs and scalp topographies. Note the similarity of waveforms and scalp topographies in the ‘reach’ and ‘withdraw’ conditions. Middle panel: Timecourse of coefficients of the intensity regressor (β_Int_) and peak scalp topographies. Note the similarity of the β_Int_ waveforms and scalp topographies in the two conditions. Bottom panel: Timecourse of coefficients of the reaction time regressors (β_RT_) and their peak scalp topographies. The lower waveform shows the *T*-values of the comparison between ‘reach’ and ‘withdraw’ β_RT_. Significance threshold (critical value, T=2.2) is shown by the red line. Note that the only region of significant difference between the two β_RT_ (highlighted in gray) falls in the N2 time window. Note also the dissimilar scalp topography of the β_RT_ in the two conditions.

**
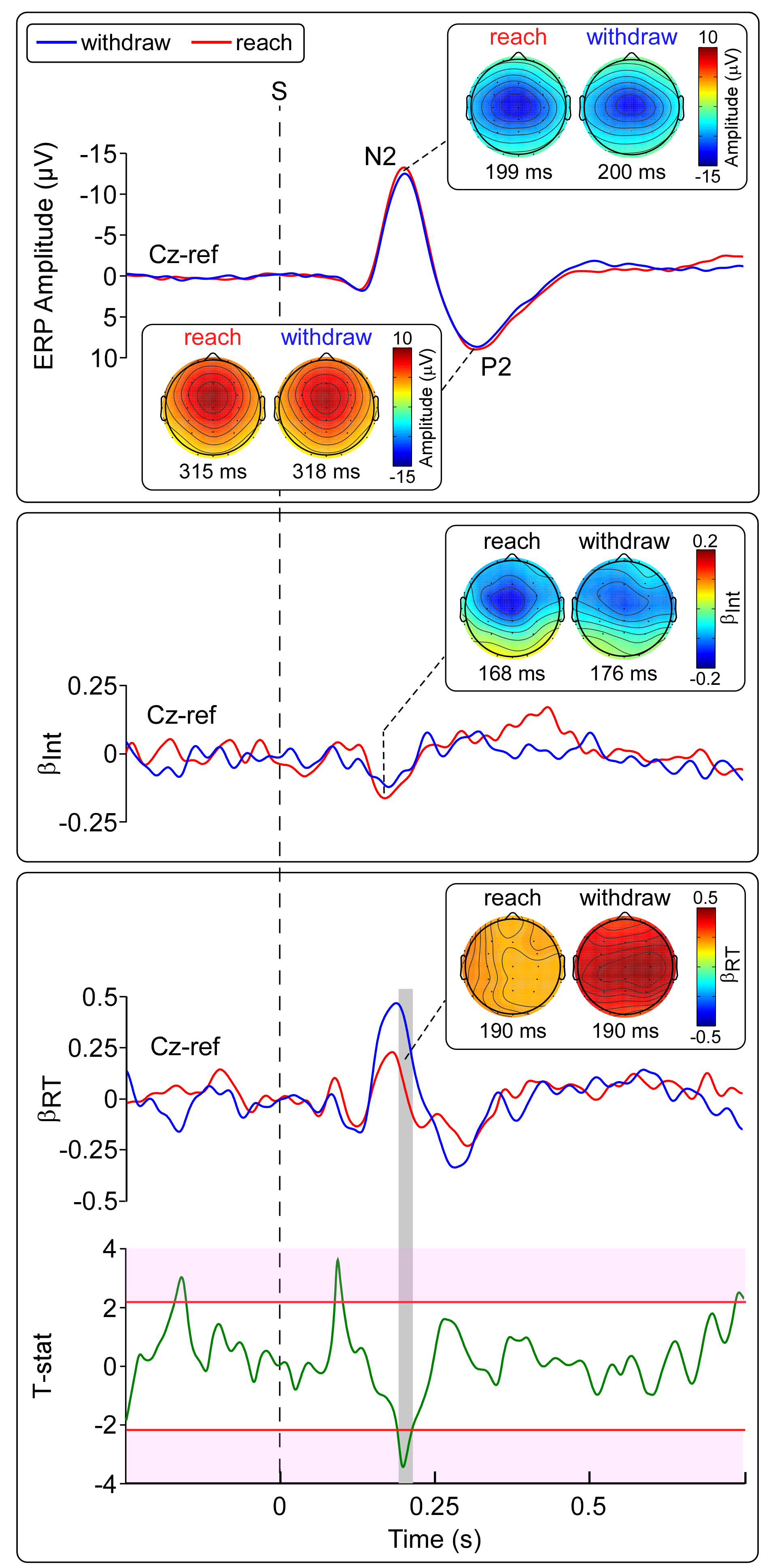
**

**Figure S3. Experiment 2. LEP waveforms and multiple linear regression results of reaction time dataset (n=22/22).** Top panel: Group-level LEPs and scalp topographies. Note the similarity of waveforms and scalp topographies in the ‘punishment’ and ‘control’ conditions. Middle panel: Timecourse of coefficients of the intensity regressor (β_Int_) and peak scalp topographies. Note the similarity of the β_Int_ waveforms and scalp topographies in the two conditions. Bottom panel: Timecourse of coefficients of the reaction time regressors (β_RT_) and their peak scalp topographies. The lower waveform shows the *T*-values of the comparison between ‘punishment’ and ‘control’ β_RT_. Significance threshold (critical value, T=2.08) is shown by the red line. Note that the only region of significant difference between the two β_RT_ (highlighted in gray) falls in the N2 time window. Note also the reduced amplitude in the scalp topography of the β_RT_ for ‘control’ condition compared to the ‘punishment’ condition.


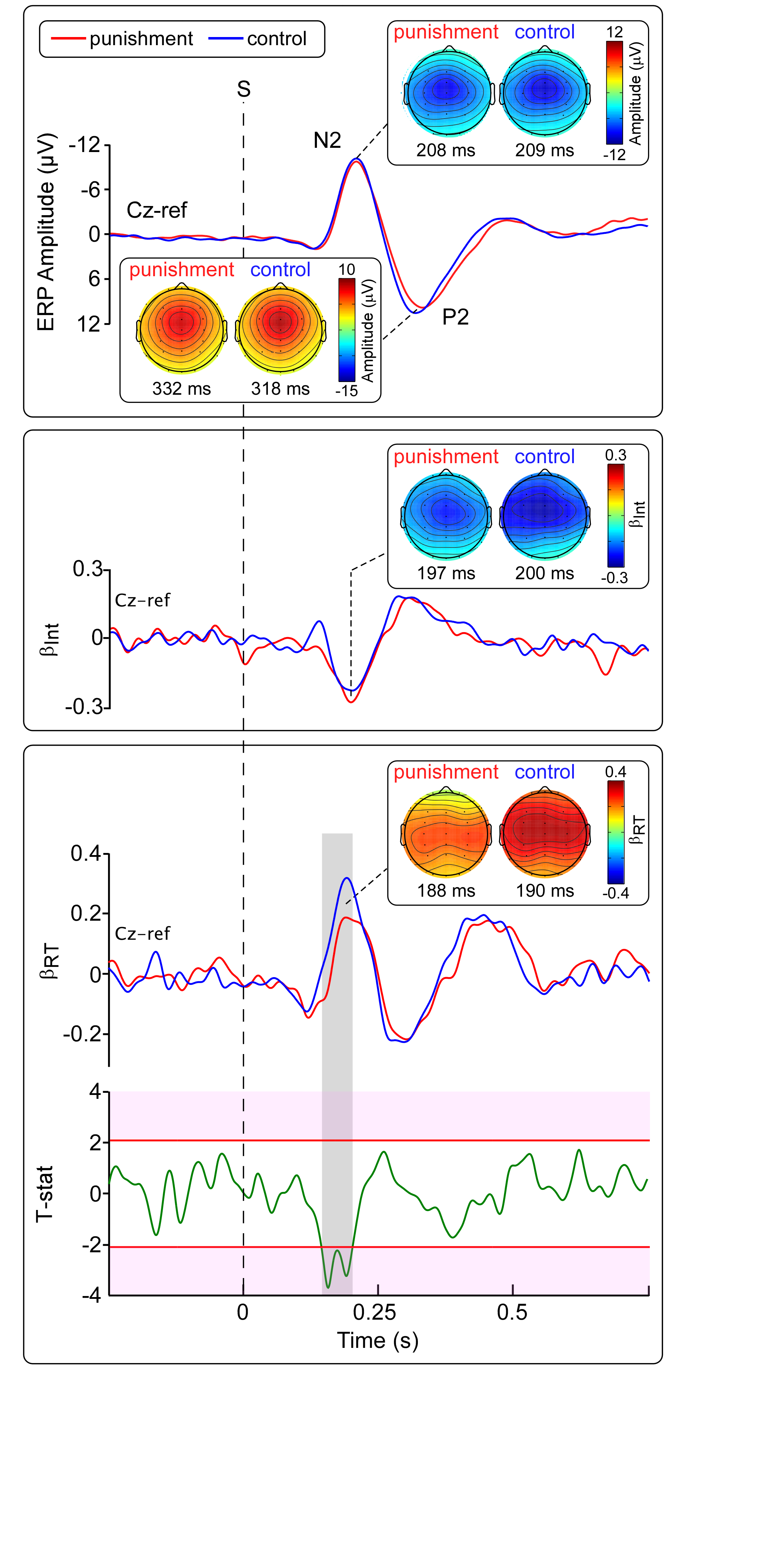

Supplement: Supplementary Data [file supp_bhv149_bhv149supp.docx]
